# Development and Validation of a Custom-Built System for Real-Time Monitoring of In Vitro Rumen Gas Fermentation

**DOI:** 10.3390/ani15152308

**Published:** 2025-08-06

**Authors:** Zhen-Shu Liu, Bo-Yuan Chen, Jacky Peng-Wen Chan, Po-Wen Chen

**Affiliations:** 1 Department of Safety, Health and Environmental Engineering, Ming Chi University of Technology, New Taipei City 24301, Taiwan; zsliu@mail.mcut.edu.tw (Z.-S.L.); jackynike061548@gmail.com (B.-Y.C.); 2 Chronic Diseases and Health Promotion Research Center, Chang Gung University of Science and Technology, Chiayi 61363, Taiwan; 3 Department of Veterinary Medicine, College of Veterinary Medicine, National Chung Hsing University, Taichung 40249, Taiwan; pwchan@nchu.edu.tw

**Keywords:** in vitro fermentation, artificial rumen systems, climate greenhouse gas, methane, gas production

## Abstract

While the Ankom RF system facilitates efficient high-throughput in vitro fermentation studies, its high cost and limited flexibility constrain its broader applicability. To address these limitations, we developed and validated a low-cost, modular gas monitoring system (FerME), assembled from commercially available components. To evaluate its performance and reproducibility relative to the Ankom RF system (Ankom Technology, Macedon, NY, USA), in vitro rumen fermentation experiments were conducted under strictly controlled and identical conditions. Whole rumen contents were collected approximately 2 h post-feeding from individual mid- or late-lactation dairy cows and immediately transported to the laboratory. Each fermenter received 50 mL of processed rumen fluid, 100 mL of anaerobically prepared artificial saliva buffer, and 1.2 g of the donor cow’s diet. Bottles were sealed with the respective system’s pressure sensors, flushed with CO_2_, and incubated in a 50 L water bath maintained at 39 °C. FerME (New Taipei City, Taiwan) and Ankom RF fermenters were placed side-by-side to ensure uniform thermal conditions. To assess the effect of filter bag use, an additional trial employed Ankom F57 filter bags (Ankom Technology, Macedon, NY, USA; 25 μm pore size). Trial 1 revealed no significant differences in cumulative gas production, volatile fatty acids (VFAs), NH_3_-N, or pH between systems (*p* > 0.05). However, the use of filter bags reduced gas output and increased propionate concentrations (*p* < 0.05). Trial 2, which employed filter bags in both systems, confirmed comparable results, with the FerME system demonstrating improved precision (CV: 4.8% vs. 13.2%). Gas composition (CH_4_ + CO_2_: 76–82%) and fermentation parameters remained consistent across systems (*p* > 0.05). Importantly, with 12 pressure sensors, the total cost of FerME was about half that of the Ankom RF system. Collectively, these findings demonstrate that FerME is a reliable, low-cost alternative for real-time rumen fermentation monitoring and could be suitable for studies in animal nutrition, methane mitigation, and related applications.

## Simple Summary

We developed a low-cost and easy-to-build device to monitor gas production during fermentation, such as in cow stomachs. It performed similarly to a commercial system but gave more consistent results. We also found that encasing the feed in filter bags reduced gas production, likely due to limited microbial access. This tool provides an affordable and flexible option for fermentation studies in animal science and environmental research.

## 1. Introduction

In vitro cumulative gas production measurement or pressure-based headspace (the space in the fermentation bottle excluding the liquid volume) gas detection has become a widely used approach for studying various rumen fermentation and food fermentation processes since its initial introduction [1,2]. The original technique relied on determining gas volume by manually tracking the displacement of a plunger in a graduated syringe [1], a method that is both time- and labor-intensive [3]. To address these limitations, several semi-automated and fully automated systems have been developed [4,5,6], which monitor gas accumulation in the headspace of closed fermentation bottles. Notably, previous studies have demonstrated that rapid gas buildup—particularly during the early phase of active fermentation—can interfere with gas release from the buffered rumen medium, thereby inhibiting substrate degradation and reducing fermentation rates [5,7]. Consequently, appropriate and timely gas sampling (pressure relief), or the use of an integrated automatic exhaust system, is critical to maintaining optimal fermentation conditions [3].

At present, various systems and modules have been developed to measure gas production in rumen and other fermentation contexts [4,5,8]. These systems differ in their applicability, advantages, and limitations. For example, the semi-continuous RUSITEC platform (the rumen simulation technique), designed to better simulate continuous dietary input and saliva buffering in the rumen, enables long-term fermentation studies that closely replicate in vivo conditions [9]. However, its complex setup and high maintenance requirements limit its routine use. In contrast, the batch fermentation-based Ankom RF system offers rapid setup and ease of operation, making it well suited for high-throughput screening and preliminary studies [10]. The latest Ankom RF system integrates pressure sensors and valves for real-time measurement of gas and headspace temperature during short-term incubations, typically within a 48 h incubation period. Its portability and modular design have encouraged widespread adoption, supported by cross-laboratory validations [11] and direct comparisons with other gas production systems [12]. It has also been successfully applied in various fields, including equine nutrition [13] and microbial metabolic studies [14]. Nevertheless, the Ankom RF module measures only gas temperature inside the serum bottle and lacks the ability to monitor the temperature or pH of the fermentation medium. An earlier study conducted a ring test across four laboratories (Italy—IT, Spain—SP, Wales—WA, and Denmark—DK) using the same wireless equipment (Ankom Technology), substrates, and laboratory protocol, and evaluated the repeatability and reproducibility of gas production parameters according to ISO 5725-2. The study reported that approximately 12.5% of the data had to be excluded from analysis due to battery failure or gas leakage [11], highlighting battery instability as a significant limitation. Consistent with this report, we have also encountered practical challenges during extended incubations in our laboratory, including wireless signal interruptions, battery-related performance fluctuations, valve malfunctions, and limited liquid sampling capacity due to the sealed design. These issues are likely exacerbated by the use of rechargeable batteries, whose output may vary with charge status, aging, or environmental conditions. In addition, placement of the RF sensors in water baths alongside serum bottles may allow water vapor intrusion, contributing further to signal instability. Lastly, according to the user manual, the Ankom RF system is rated for pressure measurements only up to 10 psi, which may limit its application in fermentation settings characterized by high gas production.

In addition to commercial systems, a few research groups have attempted to develop fully automated or custom-built platforms for in vitro gas monitoring. While these systems demonstrate advanced capabilities such as continuous methane detection and real-time data acquisition, they often involve complex integration of valves, GC analyzers, and heating units, making them less accessible for routine use in standard laboratories. These approaches highlight promising directions, but their complexity and setup requirements may pose challenges for widespread adoption [15,16,17].

While the Ankom RF system remains widely used and validated in many laboratories, the abovementioned limitations have motivated the development of complementary approaches. Our intention is not to replace existing systems, but rather to provide an alternative solution with different engineering trade-offs that may be more suitable for certain experimental applications. Accordingly, we selected the Ankom RF system as the benchmark for comparison, as it represents a widely adopted and standardized platform. Designing a system with comparable functionality allows for meaningful cross-study comparisons and broader relevance within the research community. Rather than pursuing full automation, our goal was to create a low-cost, easily assembled fermentation monitoring system that provides accurate gas production measurements along with a large-format, real-time display interface.

Nevertheless, this study aimed to explore and develop a custom-built gas production monitoring system. The objective was to evaluate alternative designs or approaches that may address some of the known limitations of existing technologies—such as data loss due to hardware malfunction—and to improve the reliability of in vitro gas production measurements. Through ongoing technical refinement, this work seeks to offer a more robust measurement platform for feed evaluation applications. To this end, we developed a custom Fermentation Monitoring Equipment (FerME) system for real-time rumen fermentation monitoring. The FerME system is fully sealed, waterproof, and compatible with standard serum bottles equipped with various sampling ports, enabling convenient gas and liquid sampling. It is constructed using widely available commercial components, making it a low-cost and user-assembled solution. The system integrates a paperless data logging module and a large-format display capable of real-time visualization for up to 12 serum bottles. While the display interface supports 12 channels, the data acquisition infrastructure is technically scalable for larger setups. The system continuously measures gas pressure, temperature, and pH, and operates on a stable 110 V power supply, avoiding reliance on battery power. The FerME module supports pressure detection up to 50 psi, exceeding the 10 psi limit of the Ankom RF system, thus broadening its applicability to high-gas-output fermentation scenarios. Additionally, the system can be equipped with an automatic pressure relief valve or configured for scheduled manual gas release, preventing excessive pressure buildup that may inhibit microbial activity and reduce fermentation efficiency. Importantly, all performance comparisons in this study were conducted under rigorously matched experimental conditions, including identical rumen fluid sources, substrates, incubation environments, and bottle types. This ensured a fair and technically valid basis for evaluating the relative strengths and limitations of both systems. Together, these features enhance FerME’s reliability, flexibility, and suitability for long-term and diverse fermentation studies.

This study aims to evaluate the performance and practical utility of the FerME system in comparison with the Ankom RF system, with a focus on reproducibility, operational convenience, and real-time data acquisition in in vitro rumen fermentation experiments. In addition to technical performance, cost-efficiency was also taken into account. A 12-channel FerME system costs approximately $6000 USD, whereas the equivalent Ankom RF system—comprising one main control unit and 12 wireless modules from the latest GEN 3 RF Gas Production System—costs over $12,297 USD. These findings provide valuable insights to help researchers select in vitro fermentation platforms that align with both their experimental objectives and budgetary constraints.

## 2. Materials and Methods

### 2.1. Animals and Rumen Fluid Collection

All animal experiments and procedures, including the collection of rumen fluid samples from dairy cows, were conducted in strict accordance with the guidelines and approvals of the Institutional Animal Care and Use Committee (IACUC) of National Chung Hsing University (protocol number 113-008). The methods employed for sample collection adhered fully to the approved protocol. Rumen fluid used in this study was obtained from two healthy, lactating Holstein cows, both 4 years old and in their first lactation. The samples were collected either via pre-existing rumen cannulas or by orogastric tubing during routine veterinary checks or educational activities at the university farm. No additional procedures were performed specifically for the purpose of this research. Since the study aimed to compare gas pressure profiles between two systems using the same rumen fluid sample, the detailed physiological background of the donor animals was not considered a critical variable. Furthermore, as only surplus rumen content was used for in vitro fermentation assays, no invasive procedures or additional handling of animals were required.

### 2.2. FerME Module Development

To provide an affordable and accessible solution for real-time gas monitoring in microbial fermentations, we developed the FerME module using commercially available, low-cost components. The system was entirely self-assembled in our laboratory, demonstrating that a fully functional and expandable pressure-monitoring platform can be constructed without reliance on proprietary or expensive instrumentation. The core of the FerME module consists of a sealed, waterproof pressure sensor head that directly connects to standard serum bottles (100–2000 mL). This setup supports flexible sampling through side ports and accommodates diverse fermentation volumes and vessel types. The sensor is compatible with long-duration fermentations and rapidly gas-producing systems, with a measurement range of 0.01–50 psi and fine resolution down to 0.01 psi.

The sensor system interfaces with a custom-built, paperless data logging module, which includes a digital display panel for real-time visualization of up to 12 channels. Although only 12 fermentation vessels can be displayed simultaneously, the underlying data acquisition system supports unlimited channel expansion, allowing high-throughput experimentation. All pressure data are automatically recorded in spreadsheet-compatible formats (.csv, .xls), streamlining downstream analysis. Additionally, users can freely configure the data recording interval, ranging from high-frequency acquisition (e.g., one data point per second) to less frequent logging (e.g., every 5 or 10 min), depending on the experimental requirements. In the current study, the pressure of the fermentation system was set to be recorded at 10 min intervals. Most importantly, the system is powered by a stable 110 V AC source, ensuring uninterrupted performance throughout prolonged incubation periods. To further safeguard against unexpected power outages, the system can be optionally connected to an uninterruptible power supply (UPS) to maintain continuous operation and data integrity. In contrast, the Ankom RF system is powered by rechargeable batteries, which may lead to issues such as limited battery life or sudden power loss during extended incubations.

The FerME module allows for modular upgrades, including optional integration with temperature and pH sensors and solenoid-controlled exhaust valves for automated gas release. By leveraging do-it-yourself (DIY) assembly and cost-efficient components, the FerME platform offers a scalable and customizable alternative to commercial fermentation monitoring systems. Although primarily developed for in vitro rumen fermentation, its flexible design may permit adaptation to other gas-producing in vitro fermentation or digestion studies, such as silage or hindgut simulations. However, the suitability of the system for these applications would require further validation, as gas production kinetics and detection sensitivity may differ across experimental contexts.

### 2.3. In Vitro Fermentation

In vitro fermentation was conducted with minor modifications based on previously reported methods [5,18]. Whole rumen contents were collected approximately 2 h post-morning feeding from individual cows, transported to the laboratory in prewarmed, sealed flasks, and immediately homogenized. The rumen fluid was obtained from two healthy, lactating Holstein cows fitted with rumen cannulas, maintained on a standard total mixed ration (TMR) at the university farm. These animals were regularly used for teaching and research purposes under approved institutional guidelines, and no additional interventions were conducted for this study. The rumen fluid was filtered through two layers of cheesecloth to separate liquid from residual solids prior to incubation [19]. Fermentations were conducted using either the Ankom RF gas production system (Ankom Technology Corp., Fairport, NY, USA) or the custom-designed FerME module. Ground substrate, identical to the donor cow’s diet and passed through a 1 mm sieve, was weighed at 1.2 g (same ration fed to donor cow) and placed into 250 mL Ankom bottles. Artificial saliva was prepared under anaerobic conditions as previously described [20]. Subsequently, 50 mL of prepared rumen fluid and 100 mL of anaerobically prepared artificial saliva buffer were added as described previously [21]. For the Ankom RF module, bottles were sealed with the system’s pressure sensor modules, flushed with carbon dioxide prior to incubation, and placed in a 50 L water bath (BT-680D, YIH DER, New Taipei City, Taiwan) preheated to 39 °C. Incubations lasted 48 h with five replicates per treatment.

For the FerME module, the same rumen fluid, artificial saliva, and substrate preparations were used as in the Ankom RF system. Fermentation was conducted in standard 250 mL serum bottles sealed with the FerME gas detection head and incubated in a gently shaking water bath under identical temperature conditions. All serum bottles from both the FerME and Ankom RF systems were placed side-by-side in the same 39 °C water bath to ensure uniform thermal conditions. To eliminate variability across systems, the same feed substrates, rumen fluid preparations, and buffer solutions were used in all experimental groups. Finally, to assess the impact of using filter bags on fermentation outcomes, we conducted an additional fermentation trial using Ankom filter bags (F57; 25-micron pore size).

### 2.4. Sampling and Gas Evaluation

Real-time gas production data from both modules were recorded at five-minute intervals via pressure sensors interfaced with a computer. Headspace gas samples were collected from each fermenter at 24 and 48 h using sealed gas syringes for methane and carbon dioxide analysis. After 48 h, fermentation bottles were opened and pH was measured immediately using a calibrated glass electrode pH meter.

Methane and carbon dioxide concentrations were quantified by gas chromatography (8610C; SRI Instruments, Torrance, CA, USA) equipped with a ShinCarbon packed column (ST 80/100, 2 mm ID). To ensure accurate and reliable gas quantification, the gas chromatograph was conditioned prior to each analytical session, including a one-hour oven warm-up at 230 °C. Standard calibration curves for CH_4_ (5–25%) and CO_2_ (40–80%) were generated using certified gas cylinders and mass flow controllers, yielding R^2^ values ≥ 0.997. Instrument performance was routinely verified by reanalyzing low, medium, and high concentration quality control samples within the calibration range. Standard curves were updated at least once per month or whenever deviations in QC performance were observed. Calibration gases were freshly prepared using a mass flow-controlled dilution system with nitrogen as the carrier gas.

### 2.5. Volatile Fatty Acid (VFA) Analysis

VFAs were analyzed via high-performance liquid chromatography (HPLC; KNAUER-AZURA, Berlin, Germany). Fermentation fluid samples were mixed with 25% (*w*/*v*) metaphosphoric acid (1:5 ratio) to precipitate proteins, shaken vigorously for 5 min, centrifuged at 12,000× *g* for 15 min at 4 °C, and the supernatants stored at −20 °C until analysis [22]. Separation was performed using a SunShell RP-AQUA C28 column (ChromaNik Technologies Inc., Osaka, Japan) with 0.01% trifluoroacetic acid in water as the mobile phase, and detection at 210 nm. Acetic, propionic, and butyric acids were quantified simultaneously.

### 2.6. Dry Matter Digestibility

Dry matter digestibility (DMD%) was evaluated to quantify the extent of substrate degradation during in vitro rumen fermentation as described previously [23]. At the beginning of the experiment, 1.2 g of dried and ground feed substrate (passed through a 1 mm sieve) was accurately weighed into individual Ankom F57 filter bags. Each bag was sealed and recorded as the initial dry weight. Following 48 h of in vitro incubation, the filter bags were carefully removed from the fermenters and gently rinsed with running tap water until the rinse water was clear, to remove residual rumen fluid and unattached microbial biomass. The washed bags were then oven-dried at 60 °C in a forced-air oven for at least 48 h, or until a constant weight was achieved. Blank filter bags (without substrate) underwent the same procedure and were used to correct for bag weight changes during incubation and drying.

Dry matter digestibility was calculated using the following formula:

DMD (%) = [(Initial dry weight − Final dry weight)/Initial dry weight] × 100

All samples were analyzed in triplicate, and values were corrected using the average weight change observed in blank bags to ensure accuracy.

### 2.7. Ammonia Analysis

Ammonia concentration was determined using the phenol-hypochlorite colorimetric method (Berthelot reaction) following established protocols [24]. Samples were appropriately diluted, reacted with phenol reagent (phenol and sodium nitroprusside), incubated with hypochlorite reagent (sodium hypochlorite and sodium hydroxide) at 37 °C for 15 min, and absorbance was measured at 630 nm using nano-spectrophotometer (SPECTROstar Nano, BMG LABTECH, Ortenberg, Germany). Each batch of rumen fluid samples was analyzed using freshly prepared calibration standards at 10, 20, 40, 60, 80, and 100 mg/dL NH_3_-N, covering the expected range of sample concentrations. The calibration curve was strictly linear within this range (R^2^ ≥ 0.998). Standard curve fitting beyond 100 mg/dL may deviate and exhibit nonlinearity, and thus such concentrations were excluded from quantification calculations. Quality control samples were re-analyzed at low, medium, and high levels within the calibration range prior to each run to verify instrumental stability. Concentrations were quantified against a standard curve prepared from ammonia standards.

### 2.8. Statistical Analysis

Data were analyzed using SPSS software (version 20.0, IBM Corp., Armonk, NY, USA). Normality of data distributions was assessed using Shapiro–Wilk tests and visual inspection of residuals and Q-Q plots. Data met the assumptions for parametric testing, supporting the use of one-way ANOVA and unpaired two-tailed Student’s *t*-tests for group comparisons. Data are presented as mean ± standard deviation (SD), and statistical significance was set at *p* < 0.05. For comparisons involving more than two groups, one-way ANOVA was followed by Tukey’s post hoc test, which adjusts for multiple comparisons to control the Type I error rate. For pairwise comparisons between two groups, unpaired two-tailed Student’s *t*-tests were used without further correction, as these were planned comparisons.

## 3. Results

### 3.1. Performance of the FerME System

**Figure 1:**
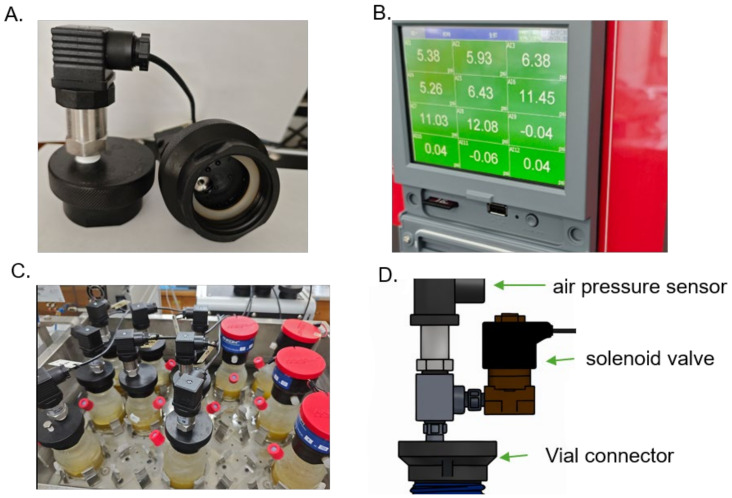
Overview of the FerME module. (**A**) Pressure sensor module connector designed for attachment to standard serum bottles (connector shown unmounted), demonstrating compatibility with various vessel volumes. (**B**) Photograph of the main display panel connected to a computer workstation, showing the touchscreen interface for system operation. (**C**) FerME and Ankom RF modules connected to serum bottles (featuring side ports for convenient gas and liquid sampling) and incubated together in a water bath for side-by-side comparison. (**D**) Computer-generated schematic illustrating the external structure and key components of the FerME module, including the air pressure sensor, solenoid valve, and vial connector; this schematic provides a visual overview of the connector shown in (**A**), without depicting internal details.

Figure 1A illustrates the basic configuration of the FerME module. This subfigure shows the pressure sensor module connector specifically designed for attachment to standard serum bottles (connector shown unmounted), supporting vessels of various volumes (100, 250, 1000, and 2000 mL). This demonstrates the system’s compatibility with conventional laboratory fermentation vessels.

In Figure 1B, a photograph of the main control panel is shown. This image provides a close-up of the touchscreen interface used to operate the system. The panel is linked to a computer workstation for automated pressure measurements at user-defined intervals. Pressure data are digitally recorded and can be exported in standard spreadsheet formats (e.g., Excel) for subsequent analysis. The display panel also facilitates real-time visualization of pressure fluctuations, enabling simultaneous monitoring of up to twelve independent fermentation conditions, thereby enhancing comparative experimental design flexibility.

Figure 1C presents the experimental setup used to validate the FerME system, where both the FerME and the commercial Ankom RF modules are connected to side-port serum bottles and incubated together in a water bath. This side-by-side comparison setup highlights the comparable usability of the wired FerME system relative to the wireless Ankom RF module. The wired design of our system does not interfere with the temperature stability of the water bath. The cables pass through small openings on the sides of the water bath lid—openings that are standard in shaking water baths—allowing the lid to close properly. Since the fermentation bottles are fully submerged in the thermostatic water, the overall temperature remains stable during operation.

The FerME system provides reliable pressure measurement while maintaining operational simplicity and ensuring easy access to data. The pressure sensor in the FerME system has a sensitivity of 0.01 psi and supports a detection range from 0.01 to 50 psi, accommodating a wide range of fermentation conditions and rapid gas production scenarios.

Figure 1D is a computer-generated schematic that illustrates the external appearance and key components of the pressure sensor connector shown in Figure 1A. It includes labeled elements such as the air pressure sensor, solenoid valve, and vial connector. This schematic provides a clearer visual representation of the core components involved in the FerME system’s pressure monitoring setup, without depicting internal structures.

Although only two independent trials are described in this report, the FerME system has been extensively tested in our laboratory across different fermentation setups, confirming its consistent performance and functional reliability (Supplementary Figure S1). These two trials were selected for formal presentation due to their representative biological variation and controlled comparison with a commercial system.

### 3.2. Gas Production Performance Compared to the Ankom RF System

**Figure 2:**
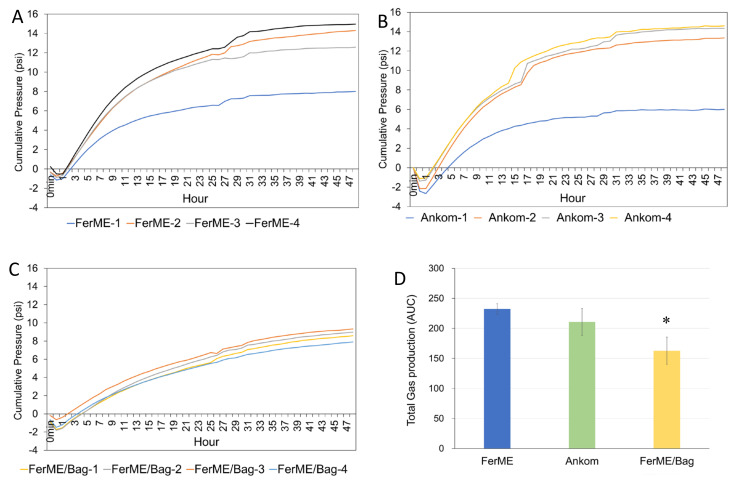
Profiles of cumulative gas pressure during in vitro rumen fermentation using different fermentation systems. (**A**) Cumulative gas production profiles from in vitro fermentation using the FerME system. (**B**) Cumulative gas production profiles from in vitro fermentation using the Ankom RF system. (**C**) Cumulative gas production profiles from in vitro fermentation using the FerME system with filter bags (FerME/Bag). Each line represents an individual replicate bottle (*n* = 4 per group). Identical rumen fluid (from a late-lactation dairy cow) and feed substrates were used across all systems, and all serum bottles were incubated simultaneously in the same 39 °C shaking water bath. (**D**) Total gas production, expressed as the area under the curve (AUC), is presented as mean ± standard deviation (SD). Asterisks (*) indicate a statistically significant difference (*p* < 0.05) compared to the FerME and Ankom RF groups, as determined by a Tukey’s HSD post-hoc test. Note: One replicate from both the FerME (FerME-1) and Ankom RF (Ankom-1) groups exhibited aberrant fermentation profiles with markedly reduced gas production; these were excluded from the AUC-based ANOVA to prevent data skew.

To assess the performance of the FerME system in monitoring gas accumulation, we compared its fermentation monitoring capacity with the commercial Ankom RF system. All fermentation reactions were conducted in 250 mL serum bottles placed in the same 39 °C shaking water bath using identical rumen fluid and dietary substrates. In the first experiment (Figure 2), conducted with rumen fluid from a late-lactation dairy cow, treatments included FerME, Ankom RF, and FerME with filter bags (FerME/Bag), each with four replicates. Total gas production profiles over 48 h were highly comparable between the FerME and Ankom RF systems. For quantitative comparison, we performed area under the curve (AUC) analysis to assess the overall gas production trends between FerME and Ankom (see Figure 2D). The AUC results indicated no significant difference between the two systems, demonstrating comparable gas pressure dynamics throughout the entire 48 h fermentation period. Moreover, although the FerME and Ankom RF results are presented in separate plots (Figure 2A,B), their gas production trends can be directly compared by evaluating pressure values at key time points (e.g., 6, 24, and 48 h) and assessing the timing and magnitude of peak gas accumulation. Visual inspection of these parameters revealed high consistency between the two systems. All curves were initially overlaid in a single panel, which further confirmed the overall similarity in gas production profiles. The final decision to display them separately was made to enhance readability and allow clearer visualization of within-group variation. However, gas production was significantly lower in the FerME/Bag group compared to the other two setups (*p* < 0.05). One replicate in both FerME and Ankom RF groups exhibited abnormal gas profiles and were excluded from statistical analysis.

**Figure 3:**
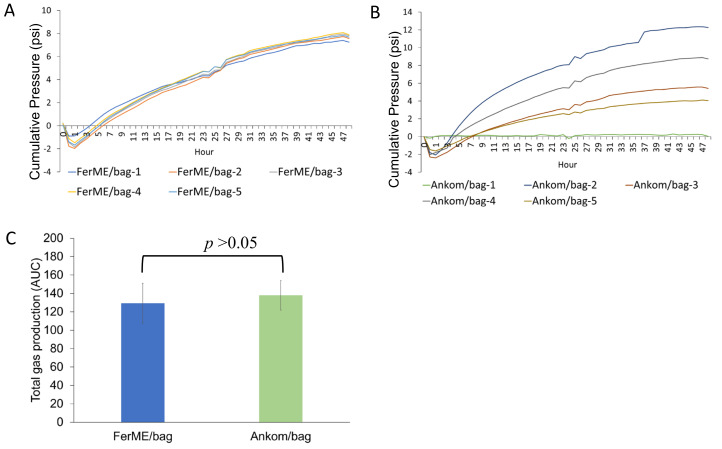
Profiles of cumulative gas pressure during in vitro rumen fermentation using the FerME/Bag and Ankom RF/Bag systems. (**A**) Fermentation using the FerME/Bag system. (**B**) Fermentation using the Ankom RF/Bag system. Each line represents an individual replicate bottle (*n* = 5 per group). Identical rumen fluid (from a mid-lactation dairy cow) and feed substrates were used for both systems, and all serum bottles were incubated side-by-side in the same 39 °C shaking water bath. (**C**) Total gas production, expressed as the area under the curve (AUC) derived from panels (**A**,**B**), presented as mean ± standard deviation (SD). No statistically significant differences were observed between the two groups (*p* > 0.05). One replicate from the Ankom RF group (Ankom-1) exhibited an aberrant fermentation profile with markedly reduced gas production and was excluded from the AUC-based ANOVA to avoid data skew.

To further validate the reproducibility of the FerME system and assess the impact of filter bag usage, a second experiment was conducted using rumen fluid obtained from a mid-lactation cow. Previous studies have shown that filter bags facilitate the measurement of amylase-treated neutral detergent fiber (aNDF) and in vitro undigested aNDF (uNDF) by eliminating the need to transfer residues from beakers to filtration crucibles [25]. Accordingly, both the FerME and Ankom RF systems incorporated filter bags in this trial, with five replicates analyzed per group. As illustrated in Figure 3A, the FerME/Bag system produced tightly clustered gas production profiles with minimal variability among replicates. In contrast, Figure 3B shows that the Ankom RF/Bag group exhibited greater variability, with one replicate (Ankom-1) demonstrating markedly reduced gas production and subsequently excluded from analysis. Total gas production, quantified as the area under the curve (AUC), was statistically indistinguishable between the FerME/Bag and Ankom RF/Bag groups (*p* > 0.05); however, the FerME/Bag system exhibited superior reproducibility across replicates. Moreover, although the difference was not statistically significant (*p* > 0.05), the effect size (Cohen’s d = 0.44) suggests a small to moderate difference between the two systems, which may be attributed to the higher variability observed in the Ankom RF/Bag group.

### 3.3. Gas Composition Analysis (CH_4_ and CO_2_)

**Figure 4:**
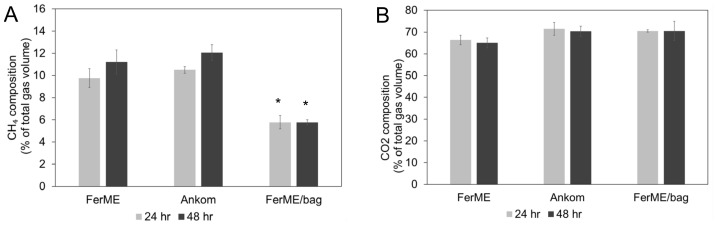
Methane (CH_4_) and Carbon Dioxide (CO_2_) Concentrations. This figure illustrates the methane (CH_4_) and carbon dioxide (CO_2_) concentrations, expressed as a percentage of total gas volume, measured at 24 and 48 h of fermentation (first trial). The gas concentrations were determined from samples harvested from the fermentation modules as shown in Figure 2, utilizing identical rumen fluid and dietary substrates. Panels (**A**) and (**B**) specifically display the concentrations of CH_4_ and CO_2_, respectively. All data are presented as mean ± standard deviation (SD). For statistical analysis, data were analyzed separately at each time point. Specifically, values from the three treatment groups at 24 h were compared only with each other, and likewise for the 48 h data. One-way ANOVA followed by Tukey’s Honestly Significant Difference (HSD) post hoc test was used to assess group differences. Differences were considered statistically significant at *p* < 0.05. Asterisks (*) indicate significant differences among treatments at the same time point.

Greenhouse gases such as methane (CH_4_) and carbon dioxide (CO_2_) are major components of ruminal fermentation gases [26,27]. Therefore, we evaluated CH_4_ and CO_2_ proportions under different experimental conditions. In the first trial (Figure 4), we assessed whether the three treatment groups differed significantly at each individual time point. For CH_4_ concentrations (Figure 4A), there were no significant differences between the FerME and Ankom modules at either 24 or 48 h (*p* > 0.05). However, the CH_4_ concentration in the FerME/Bag group was significantly lower than that of the other two groups at 24 h (*p* < 0.05), and this trend persisted at 48 h (*p* < 0.05). For CO_2_ concentrations (Figure 4B), no statistically significant differences were observed among the three groups at either 24 or 48 h (*p* > 0.05).

**Figure 5:**
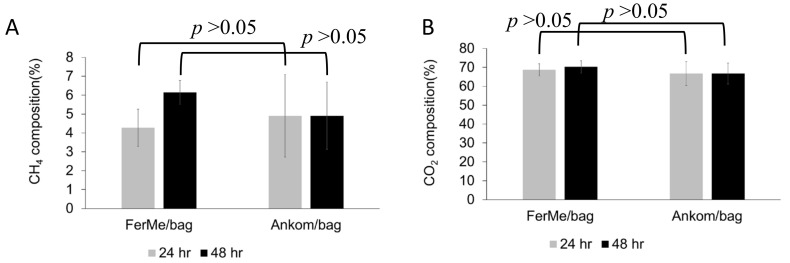
CH_4_ and CO_2_ Concentrations at 24 and 48 Hours in FerME/Bag and Ankom RF/Bag Fermentation Systems. This figure presents the CH_4_ and CO_2_ concentrations, expressed as a percentage of total gas volume, measured at 24 and 48 h during in vitro fermentation (second trial). Gas samples were collected from the FerME/Bag and Ankom RF/Bag systems, which used identical rumen fluid and dietary substrates as detailed in Figure 4. Panels (**A**) and (**B**) display the concentrations of CH_4_ and CO_2_, respectively. All data are presented as the mean ± standard deviation (SD). For statistical analysis, data were analyzed separately at each time point. Specifically, values from the two treatment groups at 24 h were compared only with each other, and likewise for the 48 h data. One-way ANOVA followed by Tukey’s Honestly Significant Difference (HSD) post hoc test was used to assess group differences. Differences were considered statistically significant at *p* < 0.05.

In the second trial (Figure 5), CH_4_ and CO_2_ proportions were compared between the FerME/Bag and Ankom RF/Bag systems at each time point independently. No statistically significant differences in CH_4_ or CO_2_ concentrations were observed between the two systems at either 24 or 48 h (*p* > 0.05). Importantly, comparisons were restricted to the same time points, and no statistical analysis was performed across time points.

Taken together, results from both trials demonstrate that the total gas production monitored by the FerME and Ankom RF systems was highly comparable. Moreover, both systems allowed for efficient gas sampling and subsequent quantification of CH_4_ and CO_2_ concentrations. Except for the condition involving the use of filter bags—which consistently showed reduced methane levels—no statistically significant differences in gas composition were observed between the two systems at matched time points. These findings confirm that the FerME module can reliably detect fermentation gas dynamics in a manner comparable to the commercial Ankom RF system.

### 3.4. Fermentation End-Product Analysis (VFAs, NH_3_-N, DMD)

Fermentation parameters were assessed at the end of each 48 h incubation. In Table 1 (first trial), rumen fluid from a late-lactation cow was used. The FerME and Ankom RF systems exhibited comparable acetate and propionate concentrations and A/P ratios (2.49 and 1.88, respectively), indicating equivalent performance in capturing microbial fermentation dynamics. In contrast, the FerME/Bag group showed elevated propionate (49.53 ± 23.06 mmol/L) and reduced acetate (31.89 ± 1.80 mmol/L), resulting in a significantly lower A/P ratio (0.64 ± 0.04).

Fermentation experiments were conducted using identical rumen fluid and dietary substrates across both the FerME and Ankom RF systems, as depicted in Figure 4. After 48 h of incubation, fermentation fluids were sampled for the quantification of volatile fatty acids (VFAs; including acetate, propionate, and butyrate, expressed in mmol/L) and ammonia nitrogen (expressed in mg/dL). Results are presented as mean ± standard deviation (SD). Statistical comparisons were performed using Student’s *t*-test, with significance defined at *p* < 0.05. The superscript “a” indicates no statistically significant difference between treatments. Asterisks (*) indicate statistically significant differences compared to the FerME and Ankom RF groups (Tukey’s honestly significant difference (HSD) test; *p* < 0.05). ND: not determined.

Table 2 presents the results from the second trial, which used rumen fluid from a mid-lactation cow. In this experiment, both FerME/Bag and Ankom RF/Bag groups utilized filter bags, allowing direct comparison of DMD and other fermentation indices under matched conditions. The two systems yielded similar values for acetate, propionate, A/P ratios (2.13 vs. 2.31), ammonia concentration, DMD (~48%), and pH, indicating high consistency between platforms.

Fermentation experiments were conducted using identical rumen fluid and dietary substrates across both the FerME and Ankom RF systems. After 48 h of incubation, fermentation fluids were sampled for the quantification of volatile fatty acids (VFAs; including acetate, propionate, and butyrate, expressed in mmol/L) and ammonia nitrogen (expressed in mg/dL). Results are presented as mean ± standard deviation (SD). Statistical comparisons were performed using Student’s *t*-test, with significance defined at *p* < 0.05. The superscript “a” indicates no statistically significant difference between treatments.

### 3.5. System Expandability and Application

The FerME module supports the integration of temperature and pH probes, enabling comprehensive monitoring of fermentation parameters. Its compatibility with serum bottles of various volumes offers experimental flexibility. An optional automatic exhaust valve is available for applications requiring controlled gas release, further expanding the system’s utility across diverse research scenarios. Nevertheless, periodic manual sampling is also commonly employed in fermentation studies as a means of pressure relief, while simultaneously allowing earlier data collection.

### 3.6. Cost and Component Comparison of FerME and Ankom RF Systems

The total cost of the FerME system is approximately USD 6000. It includes 12 ceramic diaphragm-type pressure transducers (±0.5% F.S., 0–50 psi range), 12 pressure-resistant glass fermentation vessels with sealed lids, and a touchscreen human–machine interface (HMI) featuring a 5.6-inch TFT display (640 × 480 resolution). The system is powered by a high-performance CPU (ARM Cortex-A8, 1 GHz, 256 MB RAM) and supports 12-channel analog input, expandable storage via a 16 GB BSD module, and optional modules for pH and temperature measurement. An integrated 110 V power control box with temperature monitoring is also included. The modular design enables flexible configuration and real-time digital data acquisition at a substantially lower cost.

In contrast, the total cost of the latest Ankom RF Gas Production System (GEN 3 RF Gas Production System) for a 12-channel setup is approximately USD 12,297. This base package includes five RF1X gas production modules with rechargeable batteries, five 250 mL glass bottles (four without septa ports and one with a septa port), a remote module zero, system control software, a base coordinator with USB cable, a 10-station battery charger, a valve-cleaning kit, and a positive control capsule pack. However, to expand the system to a full 12-channel setup, an additional seven gas production modules (USD 645 each), coated glass bottles with septa ports, battery extension cables, a second charger, a maintenance kit, and extra rechargeable battery packs are required, which substantially increases the total cost. The aforementioned pricing is based on the most recently published information available on the manufacturer’s official website.

Therefore, the FerME system provides a highly cost-effective and customizable alternative for laboratories that require scalable in vitro fermentation monitoring solutions without compromising data acquisition quality.

### 3.7. Statistical Validation and Data Robustness

To further address the validity of the statistical analyses, the normality of the datasets was evaluated using the Shapiro–Wilk test, along with visual inspection of residual plots and Q–Q plots. The measured variables (e.g., gas pressure, pH, and metabolite concentrations) were continuous and derived from technical replicates under controlled conditions. These assessments suggested that the data approximated normal distributions. Therefore, the use of parametric tests, including ANOVA and Student’s *t*-test, was considered appropriate based on these assumptions. Additionally, the relatively low variation among replicates indicates consistent experimental performance.

## 4. Discussion

To compare the performance and reproducibility of the newly developed FerME system with the commercial Ankom RF module, fermentation experiments were conducted under identical conditions using the same batch of rumen fluid and feed substrate. This design ensured that any observed differences in fermentation profiles could be attributed to the detection system or the use of filter bags, rather than experimental variability.

Our findings support that the pressure profiles of gas accumulation over 48 h showed that the FerME system produced fermentation curves highly consistent with those of the Ankom RF module. This consistency was further supported quantitatively by similar AUC values between the two systems, indicating that the gas detection head in the FerME system can reliably monitor gas production in real time. Importantly, the use of Ankom F57 filter bags in the FerME setup (FerME/Bag) led to significantly lower pressure readings, indicating reduced gas production. This reduction is likely due to physical limitations imposed by the filter material, which may restrict microbial access to the feed substrates, as also reported in previous studies [25,28].

Methane and carbon dioxide concentrations in the headspace gas were measured at 24 and 48 h. Both the FerME and Ankom RF systems yielded comparable CH_4_ and CO_2_ profiles, validating the accuracy of the FerME sensor in gas composition analysis. Notably, the CH_4_ percentage was inversely proportional to the CO_2_ percentage across all groups, maintaining a consistent total gas profile. The FerME/Bag group exhibited lower CH_4_ and higher CO_2_ percentages, further supporting the observation of diminished fermentation efficiency when filter bags are used.

The comparative analysis of fermentation end-products between the FerME and Ankom RF systems revealed highly consistent trends in VFA profiles, supporting the utility of the FerME system as a viable and cost-effective alternative for real-time in vitro fermentation studies. The similarity in acetate and butyrate concentrations, demonstrates that the FerME system can effectively monitor fermentation end-products and gas pressure changes under identical experimental conditions, showing comparable performance to the commercial Ankom RF module. However, since both systems only detect gas pressure and do not directly measure microbial activity, further microbiological analyses (e.g., qPCR or sequencing) are warranted in future studies to validate microbial fermentation dynamics. The distinct shift in VFA profile observed in the FerME/Bag condition, characterized by significantly elevated propionate and reduced acetate levels, likely results from limited microbial access to fiber substrates due to the physical barrier imposed by the filter bag. This interpretation aligns with previous findings indicating altered fermentation patterns under restricted substrate availability [25,28].

Collectively, these findings support the FerME system’s functional equivalence to the Ankom RF module, while also offering greater flexibility, scalability, and accessibility for laboratories seeking alternative gas measurement platforms.

Several research groups have previously attempted to develop custom-built or fully automated systems for in vitro gas fermentation monitoring [15,16,17]. For example, a fully automated batch culture platform was developed using 32 fermentation bottles, each equipped with pressure sensors and solenoid valves for total gas, hydrogen (H_2_), and methane (CH_4_) measurement. When the internal pressure reached a set threshold, the system automatically released headspace gas to a gas chromatograph (GC) for analysis. This was the first batch incubation platform capable of automated CH_4_ and H_2_ detection [16]. However, continuous nitrogen (N_2_) flushing was required to prevent carryover during GC sampling, and the system demanded time-consuming calibration procedures—such as gas volume calibration before each incubation and hourly injection of standard gases during fermentation to correct for GC drift—resulting in increased operational complexity and cost. In another study, a rumen batch fermentation platform was developed to enable continuous measurements of total gas production (GP) and methane production (MP). This system used glass bottles connected to a gas counter and an infrared (IR) gas analyzer to quantify methane levels in real time. The main advantage of this design was its ability to support continuous gas flow and kinetic analysis without pressure buildup, thereby eliminating the need for solenoid valves or pressure sensors [17]. However, due to the large headspace volume (260 mL), accurate CH_4_ quantification required precise correction for gas dynamics within the bottle. Other designs have taken a more integrated engineering approach. For instance, one system incorporated essential components such as an incubation chamber, heating units (e.g., heater fan), and temperature sensors to precisely control fermentation temperature throughout the experiment, which were built into a single housing and controlled via a unified software interface, enabling precise, automated temperature regulation throughout the experiment. The hardware and software were closely integrated, allowing automated and synchronized operation [15]. While such platforms offer excellent experimental control, they rely on complex engineering and are not easily replicated in conventional laboratory environments. In contrast to these advanced but highly specialized systems, the present study was designed with a different objective. We aimed to develop a simplified and accurate gas fermentation monitoring platform that can be self-assembled using commercially available components. The total cost of the system is approximately half that of the commercial Ankom RF system, offering a cost-effective alternative for laboratories with limited resources. Notably, we selected the Ankom RF system—a widely used and relatively simple module in fermentation research—as the benchmark for improvement. Although the Ankom RF offers ease of use and broad adoption, it is relatively expensive and presents some operational challenges. Using it as the foundation for refinement not only allowed us to address specific shortcomings (e.g., pressure limit, data loss, limited sampling), but also ensured that the performance of our FerME system could be compared directly with a widely recognized standard. A side-by-side comparison of key features between the commercial Ankom RF system and our FerME system is shown in Table 3. Rather than pursuing full automation or complex integration, our goal was to develop a reliable and user-friendly system equipped with real-time monitoring and a large-format display, thereby enhancing usability without compromising accuracy.

To further support usability without compromising accuracy, we also considered sensor calibration and reproducibility. All pressure transducers used in the FerME system were factory-calibrated (±0.5% F.S.) by the manufacturer before deployment, and users may optionally return sensors for periodic recalibration using certified pressure calibrators. Although the FerME system does not rely on proprietary calibration capsules (e.g., RF71 used in the Ankom RF system), reproducibility was verified through internal testing. Specifically, fixed volumes of air (e.g., 10 mL) were injected into sealed vessels at a constant temperature to simulate pressure changes. All twelve pressure transducers consistently registered a pressure increase of 0.45 psi, which closely matched the theoretical value calculated using the ideal gas law (PV = nRT) under the test conditions, thus confirming the high accuracy of the sensors. This procedure produced highly consistent pressure profiles across repeated trials and different modules, demonstrating good test–retest reliability under standardized conditions.

## 5. Conclusions

This study focused on the development and technical validation of a custom-built pressure monitoring module (FerME) designed for real-time in vitro gas fermentation assays. The system was assembled using commercially available components and integrated into a user-friendly interface. Validation was conducted through two independent fermentation trials using rumen fluid collected from different cows on separate dates. In both trials, FerME consistently captured fermentation pressure profiles over 48 h, with low variation across replicates.

## Figures and Tables

**Table 1:** Fermentation parameters measured after 48 h of in vitro rumen incubation under different fermentation systems (first trial).

	FerME	Ankom RF	FerME/Bag
Acetate (mmol/L)	46.31 ± 10.81 ^a^	39.7 ± 7.25 ^a^	31.89 ± 1.8 ^a^
Propionate (mmol/L)	18.59 ± 11.27 ^a^	21.14 ± 5.73 ^a^	49.53 ± 23.06 *
A/P ratio	2.49 ± 0.58	1.88 ± 0.34	0.64 ± 0.04
Butyrate (mmol/L)	8.43 ± 2.07 ^a^	10.52 ± 1.25 ^a^	9.9 ± 0.29 ^a^
NH_3_-N (mg/dL)	63.34 ± 18.02 ^a^	51.92 ± 12.82 ^a^	54.67 ± 4.88 ^a^
DMD (%)	ND	ND	45.9±2.18%
pH	5.48±0.43 ^a^	5.4±0.37 ^a^	5.95±0.04 ^a^

**Table 2:** Fermentation parameters measured after 48 h of in vitro rumen incubation between FerME/bag and Ankom/bag different fermentation systems (second trial).

	FerME/Bag	Ankom RF/Bag
Acetate (mmol/L)	31.88 ± 0.8 ^a^	33.77 ± 2.26 ^a^
Propionate (mmol/L)	14.98 ± 0.28 ^a^	14.62 ± 1.79 ^a^
A/P ratio	2.13 ± 0.05	2.31 ± 0.15
Butyrate (mmol/L)	8.41 ± 1.27 ^a^	7.75 ± 0.89 ^a^
NH_3_-N (mg/dL)	33.47 ± 5.04 ^a^	41.01 ± 19.37 ^a^
DMD (%)	49.92 ± 1.98 ^a^	50.06 ± 4.8 ^a^
pH	5.84 ± 0.02 ^a^	5.63 ± 0.33 ^a^

**Table 3:** Comparison of the FerME system and the commercial Ankom RF system.

Feature	Ankom RF System	FerME System (This Study)
Power supply	Proprietary rechargeable battery; not user-replaceable; potential risk of data loss due to power failure	110V AC power with optional UPS; enables long-term, stable operation without data loss
Pressure sensor design	Susceptible to water vapor ingress (non-sealed housing), potentially affecting data stability	Fully sealed, commercially available pressure modules; minimizes moisture-related failures
Maximum measurable pressure	Lower pressure tolerance (requires venting at ~10 psi; newer versions may improve this)	Higher pressure capacity (up to 50 psi), suitable for a wider range of experimental needs
Data display interface	Requires computer and proprietary software to view real-time pressure data	Large on-device display allows direct observation of real-time pressure values

## Data Availability

The datasets generated during and/or analyzed during the current study are available from the corresponding author on reasonable request.

## Supplementary Materials

The following supporting information can be downloaded at: https://www.mdpi.com/article/10.3390/ani15152308/s1, Figure S1: Cumulative gas pressure profiles during in vitro rumen fluid fermentation using the FerME and Ankom RF systems.
