# Supplementary figures and images for: Development and Validation of a Custom-Built System for Real-Time Monitoring of In Vitro Rumen Gas Fermentation
Source: Animals (Basel). 2025 Aug 6;15(15):2308. doi: 10.3390/ani15152308 (PMC12345559; doi:10.3390/ani15152308)

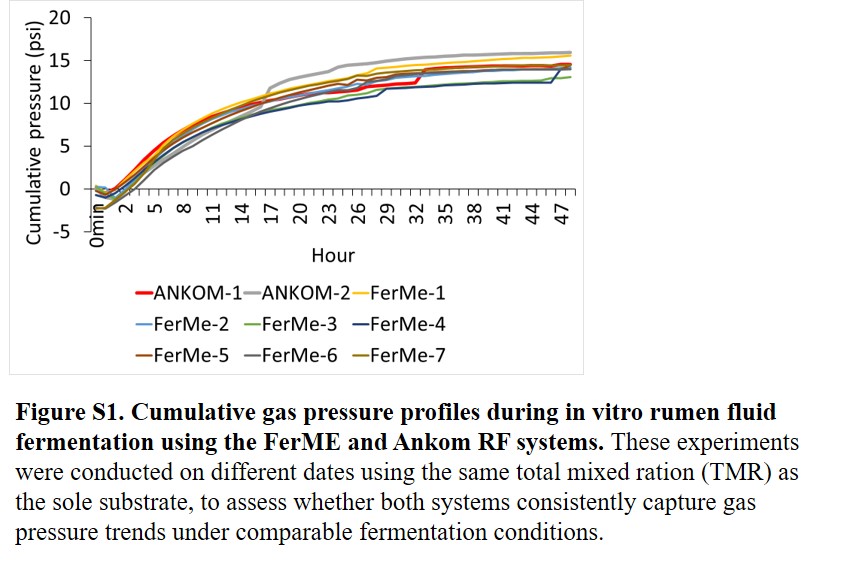

Supplement: Supplementary file 1 [file animals-15-02308-s001.zip › animals-3780869-supplementary.jpg]
